# Metagenomic Sequencing of Lloviu Virus from Dead Schreiber’s Bats in Bosnia and Herzegovina

**DOI:** 10.3390/microorganisms11122892

**Published:** 2023-11-30

**Authors:** Sejla Goletic, Teufik Goletic, Jasmin Omeragic, Jovana Supic, Naida Kapo, Melisa Nicevic, Vedad Skapur, Dunja Rukavina, Zinka Maksimovic, Adis Softic, Amer Alic

**Affiliations:** 1 University of Sarajevo—Veterinary Faculty, 71000 Sarajevo, Bosnia and Herzegovina; sejla.goletic@vfs.unsa.ba (S.G.); jasmin.omeragic@vfs.unsa.ba (J.O.); jovana.supic@vfs.unsa.ba (J.S.); naida.kapo@vfs.unsa.ba (N.K.); melisa.nicevic@vfs.unsa.ba (M.N.); dunja.rukavina@vfs.unsa.ba (D.R.); zinka.maksimovic@vfs.unsa.ba (Z.M.); adis.softic@vfs.unsa.ba (A.S.); amer.alic@vfs.unsa.ba (A.A.); 2 University of Sarajevo—Faculty of Agriculture and Food Sciences, 71000 Sarajevo, Bosnia and Herzegovina; v.skapur@ppf.unsa.ba

**Keywords:** Lloviu virus, Schreiber’s bat, pathology, sequencing, Bosnia and Herzegovina

## Abstract

Bats are a natural host for a number of viruses, many of which are zoonotic and thus present a threat to human health. RNA viruses of the family *Filoviridae,* many of which cause disease in humans, have been associated with specific bat hosts. Lloviu virus is a Filovirus which has been connected to mass mortality events in *Miniopterus schreibersii* colonies in Spain and Hungary, and some studies have indicated its immense zoonotic potential. A die-off has been recorded among *Miniopterus schreibersii* in eastern Bosnia and Herzegovina for the first time, prompting the investigation to determine the causative agent. Bat carcasses were collected and subjected to pathological examination, after which the lung samples with notable histopathological changes, lung samples with no changes and guano were analyzed using metagenomic sequencing and RT-PCR. A partial Lloviu virus genome was sequenced from lung samples with histopathological changes and found to be closely related to Hungarian and Italian virus sequences. Further accumulation of mutations on the GP gene, coding the glycoprotein responsible for cell tropism and host preference, enhances the need for further characterization and monitoring of this virus to prevent spillover events and protect human health.

## 1. Introduction

Bats (order *Chiroptera*) harbor an exceptional number of different virus species, some of which present a threat to human health. Various bat species have been identified as hosts of RNA viruses which can infect humans and cause disease, such as Hendra virus [1], Nipah virus [2], Marburg virus [3], Ebolaviruses [4], Lyssaviruses [5], Betacoronaviruses such as SARS-CoV-related viruses [6] and MERS-CoV [7]. Out of these, the family *Filoviridae* (Filoviruses) is especially significant, as most of the viruses belonging to this family infect humans and nonhuman primates, causing hemorrhagic fever that can end fatally. The most notable examples include *Orthoebolavirus zairense* (Zaire ebolavirus, abbrev. ZEBOV) and *Orthomarburgvirus marburgense* (Marburgvirus, abbrev. MARV), which can cause mortality rates in humans of up to 88% [4] and 90% [8], respectively. These two viruses are representatives of two significant Filovirus genera, *Ebolavirus* and *Marburgvirus*. A relatively new Filovirus genera has been established, *Cuevavirus,* with a sole virus species—*Cuevavirus lloviuense* (*Lloviu cuevavirus*, abbrev. LLOV). LLOV was first discovered in Spain in 2002, where a *Miniopterus schreibersii* (Schreiber’s bat or Common bent-winged bat) colony had suffered a die-off [9]. Mass mortality events in bat colonies due to bacterial, fungal and viral diseases are very rare, and most of them are attributed to either Lyssavirus infections or a fungal disease called White-Nose Syndrome (WNS) [10]. The detected LLOV in Spain marks the first Filovirus to be associated with bat colony die-offs. Since 2002, LLOV has been detected in both live and dead Schreiber’s bats in Spain [11], Hungary [12,13] and Italy [14]. Interestingly, the virus has not been detected in any other bat species, not even in the individuals of other species sharing the same roosts as affected Schreiber’s bats. The preference of LLOV for Schreiber’s bat-derived cell lines has been demonstrated in vitro [15]. The mode of transmission has not yet been elucidated, but one study found LLOV in bat flies from infected bats, suggesting the potential role of ectoparasites in passive transmission of LLOV among bats [13]. Furthermore, the virus has been successfully isolated on multiple human cell lines, as well as human macrophages [13,16], highlighting the immense zoonotic potential of LLOV. The similarities between proteins VP24 and VP35 of both EBOV and LLOV are a further cause for concern because of their mode of inhibition of interferon responses in host cells, which inhibits innate immunity [17]. Finally, increasing contact between bats and humans due to habitat loss increases the chances of a spillover event, making the Lloviu virus “an emerging disease of concern” [18]. In order to protect both bat and human health, it is essential to monitor the spread of LLOV and perform molecular studies to track virus genome evolution.

Schreiber’s bats are present in Bosnia and Herzegovina (BiH), and several sites are known to be locations of large hibernation colonies. One such site is located in an abandoned limestone mine near the town of Zvornik in eastern BiH, close to the border with the Republic of Serbia. This location is regularly monitored by bat biologists, and it hosts a colony that can number over 4000 individuals. The samples analyzed in this study were collected in April 2022 during the routine inspection of this location, which was conducted as a part of the project aiming to determine the composition of bat guano microbiome. The inspection of the site revealed that the hibernation colony sustained a die-off of approximately 110 individuals. It is worth mentioning that the same hibernation site was initially inspected a month before and at that time no dead animals were found. To date, this is the first recorded mass mortality event related to bats in BiH. To investigate the cause of the die-off, we collected dead bats and performed pathological examination as well as molecular analyses, including RT-PCR and metagenomic sequencing. This study also marks the first bat-borne virus described in BiH.

## 2. Materials and Methods

The 37 carcasses of Schreiber’s bats were collected from the hibernation site, an abandoned mine in the eastern part of BiH, in April 2022. As the animals had no apparent injuries, the selection of carcasses was randomized. The personnel collecting the samples wore personal protective equipment while on site. The carcasses were placed in separate plastic bags with ziplocks and transported in coolers containing ice packs to the University of Sarajevo—Veterinary faculty for necropsy and subsequent molecular analyses. Any ectoparasites found on the bats were collected in 2 mL Eppendorf tubes and transported in coolers, separately from the bat carcasses. Apart from the carcasses, bat guano, located in the same place as the dead bats, was also collected for molecular analyses.

At necropsy, no external lesions were observed on the bats. All internal organs were grossly examined and sampled for histopathology. The samples were fixed overnight in 10% neutral buffered formalin, trimmed and processed through different concentrations of alcohols and xylol (Microm STP 120, Tarragona, Spain), embedded in paraffin and cut at 4 to 5 μm on a microtome (Leitz Wetzlar 1400, Wetzlar, Germany). The sections were stained with hematoxylin and eosin and examined under a light microscope (BX 51, Olympus, Hamburg, Germany).

Nine lung samples with significant histopathological changes, three lung samples with no discernible changes and three guano samples were selected for molecular analysis. Tissue samples were weighed, and 20 mg of tissue was excised from each to be treated with proteinase K following the DNA Purification from Tissues protocol of QIAamp DNA Mini Kit (Qiagen, Hilden, Germany). Proteinase K treatment was performed on a shaking thermomixer at 56 °C for one hour, after which the samples were centrifuged at 12,000 rpm for 2 min and subsequently filtered using a 0.45 µm sterile filter (Milipore, Burlington, VT, USA). All the procedures were performed in a biosafety level 2+ (BSL 2+) Laboratory for molecular-genetic and forensic investigations of University of Sarajevo—Veterinary faculty, which is accredited according to the ISO/IEC 17025:2018. The filtrates were then used for the rest of the extraction procedure, which was performed according to the manufacturer’s instructions. The guano samples consisted of three guano pellets each, and they were treated with 600 µL PBS, vortexed until homogenized and then centrifuged at 12,000 rpm for 2 min. The supernatant was filtered using a 0.45 µm sterile filter (Milipore, Burlington, VT, USA). The rest of the extraction was performed with the QIAamp Viral RNA Mini kit (Qiagen, Hilden, Germany) according to the manufacturer’s instructions. The extractions were stored at −20 °C until further analysis, and the remaining tissue samples were stored at −80 °C.

In order to perform a more detailed molecular analysis, metagenomic sequencing was performed using a Sequence-independent single-primer amplification (SISPA) on a Nanopore sequencing platform. SISPA consists of two rounds (A and B). SISPA has been previously described in the literature as a strategy for the sequencing of RNA viruses [19,20]. Round A uses the FR26RV-N primer (5′-GCCGGAGCTCTGCAGATATCNNNNNN-3′) to generate cDNA. The first mix consists of 11 µL of the extraction, 1 µL 50 µM FR26RV-N primer and 1 µL of 10 mM dNTP Mix (Invitrogen, Waltham, MA, USA), after which the reaction is incubated for 5 min at 65 °C and then placed on ice for 1 min. The second mix of Round A consists of 4 µL of 5x SSIV Buffer, 1 µL of SuperScript IV Reverse Transcriptase (200 U/µL), 1 µL of 100 mM DTT and 1 µL of RNase OUT Recombinant RNase Inhibitor (Invitrogen, Waltham, USA). The first and second mixes are then combined and incubated on a thermocycler using the following conditions: 10 min at 23 °C, 50 min at 50 °C and 10 min at 80 °C. Next, 1 µL of 3′-5′ Klenow polymerase fragment (NEB, Ipswich, MA, USA) was added to the combined mix and incubated for 60 min at 37 °C and 10 min at 75 °C. Round B uses the FR20RV primer (5′-GCCGGAGCTCTGCAGATATC-3′) to amplify cDNA, the product of Round A. The reaction mixture consisted of 25 µL of PfuUltra II Hotstart PCR Master Mix (Agilent Technologies, Santa Clara, CA, USA), 1 µL of 10 µM FR20RV primer, 21 µL of RNase-free water and 3 µL of cDNA. This mix was incubated on the thermocycler according to the manufacturer’s instructions, the only change being that the annealing temperature was set to 65 °C. The final products of Round B were quantified using the Qubit dsDNA High Sensitivity Assay (Thermo Fisher Scientific, Waltham, MA, USA) for Qubit 4 fluorometer, and only those samples that exceeded the minimum concentration of 2 ng/µL were used in downstream analysis. Metagenomic sequencing was performed on a MinION Mk1C using the PCR Barcoding Kit SQK-PBK004 (Oxford Nanopore Technologies, Oxford, UK) on an R9.1.4 flow cell according to the manufacturer’s instructions.

The sequencing run lasted 26 h, during which the high-accuracy basecalling was performed in real time through integrated Guppy software v.4.2.2 in MinKNOW v.20.10.3 (MinKNOW Core v.4.1.2). Initial Q score was set to 7 during the run. Demultiplexing was also conducted in MinKNOW, and quality control was performed with FastQ Control Experiment workflow in EPI2ME v.3.6.2. From this point forward, only the reads with a Q score of ≥11 were used for further analysis. The adapters were trimmed using Porechop v 0.2.4 (https://github.com/rrwick/Porechop (accessed on 16 February 2023)), and the primers used during SISPA were trimmed using primer-chop v 1.3.2 (https://gitlab.com/mcfrith/primer-chop (accessed on 16 February 2023)). Fastq WIMP (human + viral) v2021.11.26 in EPI2ME v.3.6.2 was used for initial metagenomic analysis. Following that, we used INSaFLU (https://insaflu.insa.pt/ (accessed on 20 May 2023) [21], a web-based bioinformatic suite which includes the TELEVIR module for the identification of virus sequences in metagenomic samples by using Krakenuniq, Kaiju, FastViromeExplorer and Centrifuge for read classification. All four were used for read analysis, and the results obtained were compared to the results of the FastqWIMP analysis. The reads were then mapped to a reference sequence (NCBI accession number: NC_016144.1) using the Long Read Support tools in Qiagen CLC Genomics Workbench v23.0.4, and contigs representing the partial sequences of the LLOV genome were extracted from the mapped reads. Contigs were analyzed with BLAST to determine the sequences with the highest homology and sequence identity (https://blast.ncbi.nlm.nih.gov/Blast.cgi (accessed on 21 May 2023)). The sequences of all available complete LLOV genomes were downloaded from NCBI and used to generate phylogenetic trees in MEGA X v 10.2.4 [22]. To generate the tree, we used a Maximum Likelihood method (Hasegawa–Kishino–Yano substitution model, 1000 bootstrap replicates). The *Orthomarburgvirus* sequence was used as an outgroup.

To confirm the results of the metagenomic sequencing, a nested RT-PCR was used to amplify the Filovirus-specific L gene, as described elsewhere [23]. This gene codes for RNA-dependent RNA polymerase and is conserved across *Filoviridae* [24]. The amplicons were then sequenced using the Native Barcoding kit EXP-NBD104 with Ligation Sequencing Kit SQK-LSK109 (Oxford Nanopore Technologies, Oxford, UK) on an R9.1.4 flow cell according to the manufacturer’s instructions. The sequencing run lasted for 12 h with a Q score set to 9. Demultiplexing and trimming of the adapters and primers were performed as described above. The reads were then mapped to a reference sequence (NCBI accession number: NC_016144.1) in Qiagen CLC Genomics Workbench v23.0.4, and contigs representing the partial sequences of the Filovirus L gene were analyzed with BLAST. A phylogenetical tree was created in MEGA X v 10.2.4. with the same settings as for the LLOV sequences obtained with metagenomic sequencing.

## 3. Results

### 3.1. Pathology

Gross examination of internal organs revealed mild to moderate red discoloration of the lung, and in two individuals, the lungs were heavy and sunk upon placement in formalin. In all bats, the spleens were slightly enlarged and dark red.

**Figure 1:**
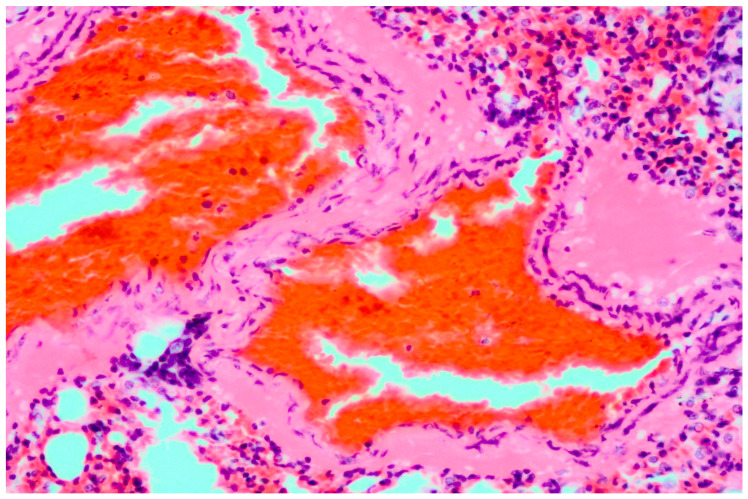
Bat lung: bright eosinophilic homogenous material is present in the arterial wall and the perivascular space. H&E 20×.

**Figure 2:**
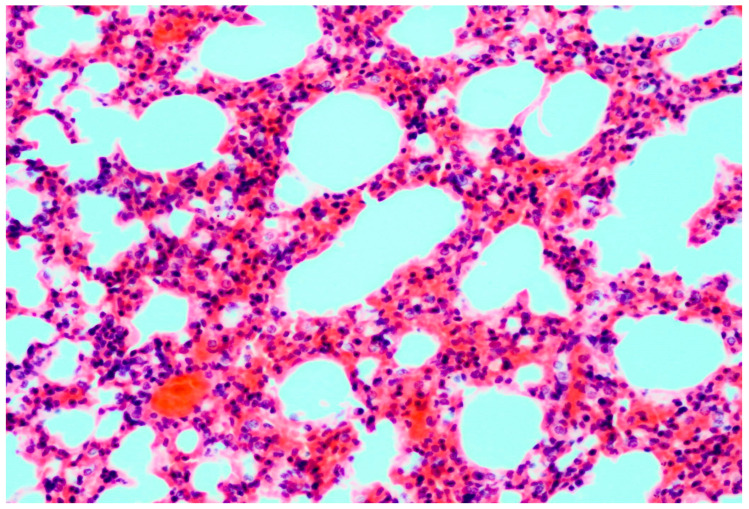
Bat lung: moderate expansion of the alveolar septa with mixed inflammatory infiltrate. H&E, 20×.

Histopathology of the lung tissue revealed diffuse congestion along with multifocal to coalescing areas of alveoli filled with moderate numbers of extravasated erythrocytes (hemorrhage) and bright eosinophilic material (edema). In multiple animals, moderate amount of bright eosinophilic, homogenous material, admixed with erythrocytes, was occluding the lumen of the large arteries. The same material was present in the interstitium of the arterial wall and was further extending into the perivascular space (Figure 1). Alveolar septa were expanded with mild to moderate numbers of mixed population of lymphocytes, macrophages and neutrophils (Figure 2). Mild to moderate infiltrates of neutrophils and small numbers of eosinophils were also present in the alveoli. In five cases, multifocal peribronchal follicular aggregates of moderate numbers of lymphocytes were observed, along with moderate hyperplasia of peribronchal glands. In two cases, small foci of alveoli were filled with small numbers of foamy macrophages. In the spleen of all animals, moderate congestion of the parenchyma was present.

### 3.2. Sequencing and Phylogenetic Analysis

The metagenomic sequencing run generated 1,150,521 of raw reads, and quality control in EPI2ME determined the average quality score as 11. After filtering the reads by quality, 672,189 reads with a Q score ≥ 11 were retained and used for further analysis. Fastq WIMP classified 278,332 reads as virus reads, and out of these, 68,749 (24.7%) were classified as *Filoviridae*; this was also the family with the highest number of classified reads (Table 1). Furthermore, within the family *Filoviridae*, an almost equal number of reads were classified into *Cuevavirus* and *Ebolavirus* genera.

**Figure 3:**
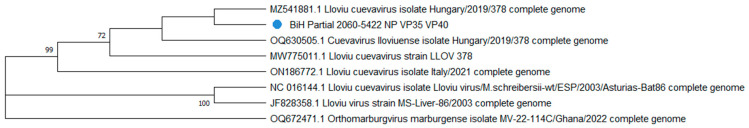
Phylogenetic tree of the BiH partial sequence of NP VP35 and VP40 genes (marked with a blue dot). The bootstrap consensus tree was constructed in MEGAX using the Maximum Likelihood method (Hasegawa–Kishino–Yano substitution model, 1000 bootstrap replicates). The *Orthomarburgvirus* sequence was used as an outgroup.

**Figure 4:**
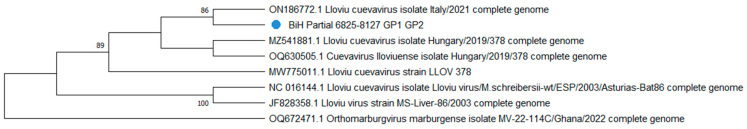
Phylogenetic tree of the BiH partial sequence of GP gene (marked with a blue dot). The bootstrap consensus tree was constructed in MEGAX using the Maximum Likelihood method (Hasegawa–Kishino–Yano substitution model, 1000 bootstrap replicates). The *Orthomarburgvirus* sequence was used as an outgroup.

The TELEVIR module of INSaFLU obtained results concordant with the results of Fastq WIMP: the highest number of viral reads (20,621) was classified as LLOV. The coverage of the genome was not high (26.35%), but the depth of coverage was determined as 907×. It’s important to note that all *Filoviridae* sequences were only detected in the nine samples with notable histopathological changes. Filovirus sequences were not found in guano or the other lung samples. Based on this, we mapped the reads to the LLOV reference sequence (NCBI accession number: NC_016144.1) and extracted two contigs. One contig mapped to the reference sequence on positions 2060–5422, thus partially covering the NP gene, completely covering the VP35 gene and partially covering the VP40 gene. The second contig mapped to the reference sequence on positions 6825–8127, which partially covered the GP gene. There was a moderate number of reads which mapped on other parts of the reference genome, but the coverage of these sections was insufficient to extract a high-quality contig. The phylogenetic analysis of the first contig revealed a 99.94% sequence identity with the *Lloviu cuevavirus* isolate Hungary/2019/378 (GenBank: MZ541881.1) and 99.73% with the *Lloviu cuevavirus* isolate Italy/2021 (GenBank: ON186772.1). The clustering with other Hungarian sequences is apparent on the phylogenetic tree (Figure 3, marked with a blue dot). On the other hand, the second contig had a 99.69% sequence identity with the *Lloviu cuevavirus* isolate Italy/2021 (GenBank: ON186772.1), and 99.62% with the *Lloviu cuevavirus* isolate Hungary/2019/378 (GenBank: MZ541881.1) (Figure 4, marked with a blue dot). Both contigs have been uploaded to GenBank (accession numbers OR714919 and OR714920) and will be released upon the publication of this study.

The fragments of the L gene were successfully amplified in the nine lung samples using the nested RT-PCR method, while no amplification was detected in the guano samples and samples with no histopathological changes. The successfully amplified fragments were sequenced, and a consensus 280 bp partial sequence of the L gene (position on the reference sequence: 13331–13611) was extracted. The BLAST analysis revealed a 100% sequence identity with both the Hungarian (GenBank: MZ541881.1) and Italian sequences (GenBank: ON186772.1), and 99.29% with the Spanish sequence (GenBank: NC_016144.1).

## 4. Discussion

In this study, we report that the LLOV has been connected to yet another mass mortality event in bats, spreading further along the area of distribution of Schreiber’s bats in Europe. In northeastern Hungary, several of mass mortality events in Schreiber’s bat colonies were recorded between 2013 and 2020 [12,13], while similar events were also recorded in northern Italy between 2018 and 2020 [14]. This mass mortality event in the largest colony of Schreiber’s bats in eastern BiH marks the first such event recorded in the country to date.

Furthermore, the present study provides more robust insight into the pathology of the lung tissue associated with LLOV infection in Schreiber’s bats. Lesions observed here further corroborate previous, very limited observations in bat die-off events from Spain [9,11]. The congestion and hemorrhage in the lung tissue, the associated edema and the interstitial pneumonia point to respiratory compromise as the most likely mechanism and mode of death. These lesions are commonly associated with viral infections, in particular Filoviruses (Ebolavirus and Marburgvirus) [4,8]. However, to demonstrate clear involvement of the LLOV in the development of the described lesions, methods such as immunohistochemistry and/or in situ hybridization should be employed.

Considering that the LLOV has only been found in Schreiber’s bats so far and is connected to the die-offs of this bat species, it was highly indicative that the same virus is also associated with this mass mortality event in BiH. However, to avoid bias, we used the metagenomic approach, which yielded partial sequences of NP, VP40, GP and a complete sequence of the VP35 gene. The metagenomic approach enabled us to study the entire lung virome of Schreiber’s bats in BiH and determine whether there are other viruses that may be relevant to the progression of the disease which caused the mass mortality. Other virus families found in lungs of the deceased bats with abundance of at least 1% in total virus reads (Table 1) were *Genomoviridae* (known to infect fungi) [25], *Coronaviridae*, *Reoviridae, Picornaviridae*, *Parvoviridae, Retroviridae* (pathogens of vertebrates) [26], *Podoviridae* (bacteriophages) [26], *Siphoviridae* (infecting bacteria) [26] and *Marseilleviridae* (infecting amoebas) [27]. Furthermore, the virus families that were found only in the lungs with pathohistological changes and not in the other lung samples were *Genomoviridae*, *Picornaviridae* and *Retroviridae*. To the best of our knowledge, the members of these virus families are not known to cause disease in bats. However, the outcome of such simultaneous infections of bats with the mentioned viral families and LLOV deserves further exploration. It is important to emphasize that no other virus families previously reported in the literature as pathogenic to bats were detected via metagenomic sequencing. Nevertheless, to rule out the known causative pathogens of mass mortality in bats, such as Lyssaviruses [28] and *Pseudogymnoascus destructans* [29], we performed PCR-based detection and found no evidence of presence of either pathogen (data not shown here). Therefore, it is highly likely that LLOV was the cause of the mass mortality event described in this work.

This conclusion is corroborated by the overwhelming presence of Filovirus reads compared to other virus reads, the pathohistological findings and the absence of other known causative agents of mass mortality in bats. Furthermore, this evidence enabled us to rule out other viruses as the causative agents of the die-off in the colony of Schreiber’s bats in BiH. This emphasizes the value of metagenomic sequencing as a tool to guide future research and discovery of new viruses. In order to study the LLOV strain detected in BiH, it is necessary to sequence the entire virus genome using an amplicon-based sequencing approach such as the one used for the sequencing of LLOV detected in Hungarian and Italian Schreiber’s bats [13].

The phylogenetic analysis of the obtained partial sequences confirms that the virus from BiH shares a somewhat higher sequence identity with Hungarian and Italian virus sequences than with those from Spain. In general, the partial sequence of the NP, VP30 and VP40 genes seems to cluster more closely with the Hungarian sequences (Figure 3), while the partial GP sequence seems to cluster more closely with the Italian sequence (Figure 4). Interestingly, even though only a partial sequence has been obtained, the highest number of point mutations was detected in the GP gene, which is known to have a high amount of variation among *Filoviridae* [30,31]. Having in mind the rate of mutation of the GP gene, it is perhaps not surprising that the partial BiH sequence clusters more closely with the Italian sequence from 2021 than with the Hungarian ones from 2019. However, the complete GP gene sequence is necessary to draw a definite conclusion. The GP gene encodes for several glycoproteins, most importantly GP_1_ and GP_2_, which form a complex that mediates Filovirus entry in host cells via the Niemann–Pick C1 (NPC1) receptor [32]. Furthermore, it seems that the GP/NPC1 interaction is an important factor in determining the tropism and host specificity of LLOV [15]. Several site-directed mutagenesis studies in vitro have shown that point mutations in the GP gene may lead to a change of virus preference from an NPC1 of one bat species to an NPC1 of another bat species, or even to a human NPC1 [15,32]. Human-derived cell lines and human macrophages have been successfully infected with LLOV in vitro, and now LLOV is considered to have a strong zoonotic potential [16]. Thus, sequencing and monitoring of the GP gene is imperative to successfully prevent human infection.

The sequenced fragment of the highly conserved region of the L gene (280 bp) was too short to be used for phylogenetic analysis, but it confirmed the presence of the LLOV in the analyzed samples.

Our findings confirm that LLOV has spread further through southeastern Europe, and it seems that the virus is spreading inside the area of distribution of the Schreiber’s bat, its only known host. It is important to note that the Schreiber’s bat is a migratory species, and the mine in the eastern BiH is a known location of a hibernation colony of this species. Come spring, these bats migrate ~107 km northeast to Petrovaradin Fortress in the Republic of Serbia to form maternity roosts. The fortress is only ~95 km from the Hungarian border, and the spread of the virus among bats might be explained by the existence of a possible migratory route between Hungary and Serbia. Banded individual bats from eastern BiH have been found in other caves in western Serbia. Furthermore, even though the Schreiber’s bat is primarily a cave-dwelling species, it may seek shelter closer to humans due to habitat loss. The abandoned mine in BiH is located behind a row of houses in the small village which is adjacent to the town of Zvornik. Petrovaradin Fortress is an important historical site, and it annually hosts a large music festival. These sites are of importance to both humans and the declining Schreiber’s bat populations, and the continuous habitat loss due to anthropological factor increases the chance of a spillover of LLOV to humans. Lloviu virus disease is already considered “an emerging disease of concern” [18], and to protect both humans and bats, it is necessary to regularly monitor known Schreiber’s bat roosting sites for mortality events, to sample live bats in order to monitor virus circulation, to regularly sequence the virus to monitor potentially harmful mutations and to reduce the number of human visits to the roosting sites to protect their habitat as well as human health.

## Figures and Tables

**Table 1:** Virus families detected in samples originating from dead Schreiber’s bats in BiH. The total number of virus reads as well as their source (lungs with or without pathological changes and guano) has been listed for each family. The virus families that are represented with less than 300 reads were not listed in the table.

Virus Family	The Total Number of Reads	Source(s) of Reads (%)		
		Lungs with Pathohistological Changes	Lungs without Pathohistological Changes	Guano
*Filoviridae*	68,749	100.00%	0.00%	0.00%
*Flaviviridae*	23,562	0.00%	0.00%	100.00%
*Microviridae*	7025	0.00%	0.00%	100.00%
*Myoviridae*	5751	0.00%	0.00%	100.00%
*Genomoviridae*	5644	10.64%	0.00%	89.35%
*Baculoviridae*	3987	0.00%	0.00%	100.00%
*Coronaviridae*	2410	20.29%	24.86%	54.85%
*Reoviridae*	2402	37.51%	46.36%	16.13%
*Podoviridae*	1743	27.71%	29.95%	42.34%
*Papillomaviridae*	1721	0.01%	0.05%	99.94%
*Picornaviridae*	1701	45.11%	0.00%	54.89%
*Iflaviviridae*	1623	0.00%	0.00%	100.00%
*Syphoviridae*	1520	26.49%	28.35%	45.19%
*Parvoviridae*	1377	42.98%	38.97%	18.05%
*Paramyxoviridae*	1068	0.13%	0.07%	99.80%
*Marseilleviridae*	906	16.27%	21.04%	62.69%
*Retroviridae*	743	18.87%	0.00%	81.13%

## Data Availability

Publicly available datasets were analyzed in this study. This data can be found here: https://www.ncbi.nlm.nih.gov/nuccore/?term=lloviu+virus (accessed on 6 September 2023). The sequence names and accession numbers are listed in Supplementary Table S1.

## Supplementary Materials

The following supporting information can be downloaded at: https://www.mdpi.com/article/10.3390/microorganisms11122892/s1, Table S1: Information about NCBI sequences used for phylogenetic analysis.
