# Supplementary material for: Metagenomic Sequencing of Lloviu Virus from Dead Schreiber’s Bats in Bosnia and Herzegovina
Source: Microorganisms. 2023 Nov 30;11(12):2892. doi: 10.3390/microorganisms11122892 (PMC10745292; doi:10.3390/microorganisms11122892)
Supplement: Supplementary file 1 [file microorganisms-11-02892-s001.zip › microorganisms-2679515-supplementary.pdf]

Supplementary Material - Metagenomic sequencing of Lloviu virus from dead bats in Bosnia and Herzegovina

Table S1. Information about NCBI sequences used for phylogenetic analysis.

Sequence name	NCBI Accession number	Submission date
Orthomarburgvirus marburgense isolate MV-22-114C/Ghana/2022, complete genome	OQ672471.1	26-JUL-2023
Cuevavirus lloviuense isolate Hungary/2019/378, complete genome	OQ630505.1	02-JUL-2023
Lloviu cuevavirus isolate Italy/2021, complete genome	ON186772.1	29-JAN-2023
Lloviu cuevavirus isolate Hungary/2019/378, complete genome	MZ541881.1	15-FEB-2022
Lloviu cuevavirus strain LLOV_378 nucleoprotein, VP35, VP40, GP1, GP2, VP30, hypothetical protein, VP24, and RNA polymerase genes, complete cds	MW775011.1	14-FEB-2022
Lloviu cuevavirus isolate Lloviu virus/M.schreibersii-wt/ESP/2003/Asturias-Bat86, complete genome	NC_016144.1	13-AUG-2018
Lloviu virus strain MS-Liver-86/2003, complete genome	JF828358.1	30-OCT-2011
